# Machining with a Precision Five-Axis Machine Tools Created by Combining a Horizontal Parallel Three-Axis Motion Platform and a Three-Axis Machine Tools

**DOI:** 10.3390/ma15062268

**Published:** 2022-03-18

**Authors:** Yuan-Ming Cheng

**Affiliations:** Department of Intelligent Robotics, National Pingtung University, Pingtung 90004, Taiwan; chengym@mail.nptu.edu.tw

**Keywords:** five-axis, OPEN CNC, parallel mechanism, three-axis platform

## Abstract

Five-axis working machines are applied in the high-precision machining of complex convex surfaces. Therefore, this study integrated a horizontal parallel three-axis motion platform and a three-axis machine tools to create a reconfigurable precision five-axis machine tools (RPFMT). A DELTA OPEN computer numerical control controller was used as the control system architecture. A human–machine interface and programmable controller were incorporated into the developed tool to achieve automatic online measurement. A suitable cutting tool was selected to calculate the five-axis NC machining code for a complex convex surface. The NC codes were input into the LabVIEW software for five-axis postprocessing conversion. A concave workpiece was cut through rough and finishing machining to verify the accuracy of the produced RPFMT.

## 1. Introduction

With the increasing complexity and diversification of products, five-axis machining technologies have been widely applied in the precision machining industry. Five-axis machine is a numerically controlled machining technique that can be used to machine complex surfaces. The five axes in the aforementioned technique are three axes of linear motion (the *X*-axis, *Y*-axis, and *Z*-axis) and two axes of rotary motion (the *A*-axis and *B*-axis, *B*-axis and *C*-axis, or *A*-axis and *C*-axis). Five-axis machine tools can achieve high tolerances for the machining of complex geometric shapes. Therefore, five-axis machining technologies have the following advantages: (1) low processing cost and time consumption; (2) one-time clamping, minimal positioning error, and high-precision surface machining; (3) multifunctionality and improved process continuity that saves time; and (4) capability of complex machining. Therefore, five-axis machines can be applied in relevant precision machining industries, such as the energy industry, aerospace, automotive, mold, machine tools, and shipbuilding industries.

Because of their advantages of high stiffness, high precision, low inertia, and simplicity, parallel mechanisms are suitable for application in machining tools. Therefore, Tsai [1,2,3] simplified a six-axis parallel mechanism into a three-axis platform and compared the features of three mechanisms, namely 3-RUU (revolute-universal-universal), 3-UPU (universal-prismatic-universal), and 3-PUU (-prismatic-universal-universal). The actuation axes of three-degree-of-freedom (DoF), three-axis parallel platforms can have three arrangements: a (1) vertical arrangement [4], (2) parallel arrangement (with parallel rails) [5], and (3) horizontal arrangement on the base platform (with intersecting rails) [6]. These three-axis platforms possess the advantages of parallel mechanisms but have a relatively large working space and high sensitivity. Therefore, in this study, a horizontal parallel three-axis motion platform and a three-axis machine tools were integrated to construct a reconfigurable precision five-axis machine tools (RPFMT). The horizontal three-axis platform manipulates the *Z*-direction as well as the values of the *α* and *β* angles.

In order to simplify the complexity of the actuation axes of the three-axis platform, these axes are arranged horizontally on the base. The axial lines intersect at the circular base center, and changing of movable platform attitudes (*Z*, *α*, *β*) is achieved through the forward and backward motions of the three actuation axes. The mechanism of the horizontal parallel three-axis motion platform (3-PRS) consists of three (two DoF) universal joints combined with a bearing. This combination enables the construction of a three-DoF joint connected to the upper (moving) platform and one-DoF joints of the three rotary axes connected to the lower (fixed) platform. The connecting rod linked to the upper and lower platforms is designed to have a fixed configuration to allow for easy disassembly and length alteration for changing the size of the working space for mechanism motion. Compared with a mechanism in which the actuation axes are used as connecting rods, the 3-PRS used in this study has a simpler design, reduces machining cost as well as machining and assembly errors, and enhances the control precision of actuation axes. Cheng et al. [7] used a laser displacement meter to measure the platform attitude. The error values of the upper platform’s origin were examined, the height values of three-axis connection points were calibrated, and the inverse kinematics was revised for improving the precision of the obtained platform attitude values, which were applied for investigating concentric boring through multiple surfaces at different angles.

Cheng and Lin [8] used two PCI-8134 four-axis servo control cards to control a reconfigurable five-axis machine tools (RFMT). One card controlled a 3-PRS parallel three-axis motion platform, and the other card controlled an X–Y motion table. Although the RFMT could be controlled, factors related to the computer system caused a delay when the number of program computations was large. In [9], an OPEN computer numerical control (CNC) controller system was used to reduce the delay. A concave element was cut through rough and finishing machining to verify the precision of OPEN CNC in RFMT control [9].

The achievement of ideal machining quality requires the use of not only high-quality machines and controllers but also computer-aided design (CAD)/computer-aided manufacturing (CAM) software with powerful functions for computing the cutting trajectory on the working surface. Postprocessing is a crucial procedure in CNC machining. The primary aim of this procedure is to convert the cutting tool document generated by the CAD/CAM software into an NC-code-containing document acceptable for the machining tool. The development of a complete software program for three-axis CAM is challenging and requires repeated testing and verification. Because different machines involve different kinematics, developing software programs for five-axis machine tools, particularly hybrid working machines [10,11,12], is more challenging than is developing software programs for three-axis CAM. The key technique of five-axis machining technology is the “rotational tool center point” function [13,14]. Tool center point control ensures that the contact point among the tool center point, cutting tool, and workpiece surface remains unchanged. Moreover, the tool center point is located on the normal line of the contact point between the cutting tool and workpiece surface, and the shank rotates around the tool center point.

Zhou et al. [14] developed a postprocessor with a general machine configuration and the function of optimized tool radius compensation. They used a generalized kinematics model and various cutting tool models to verify the locations of different five-axis machine centers. Liu and Huang [15] examined the nonsingular tool path generation of TriMule, which is a five-axis hybrid robot, and observed sudden changes in the rotation of the *C*-axis and the lengths of three telescopic legs. Fu et al. [16] proposed a postprocessing and path optimization method based on a nonlinear error to improve the precision of multijoint industrial robot-based three-dimensional (3D) printing. Guo et al. [17] developed a control system designed for a new five-DoF hybrid robot manipulator and described the mechanical structure, kinematics, dynamics, and control system of the robot manipulator. For the aforementioned robot manipulator, a new robotic deburring methodology was proposed for tool path planning and process parameter control [18].

Lai et al. [19] proposed a modular method for constructing a postprocessor system for a novel hybrid parallel–serial five-axis machine tools. The proposed algorithm can be used for easily performing conversions between cutter contact path and cutter location (CL) code and can be implemented in CAD and CAM systems. He et al. [20] integrated an abrasive belt with measurement equipment to develop a series–parallel hybrid platform for conducting in situ measurements related to blade polishing. They proposed a method for calculating the normal vector of machining points and studied the kinematics of the aforementioned platform. The total machining allowance was determined by comparing the actual and ideal contour profiles of the blade.

Lee and She [21] proposed a postprocessor capable of converting CL data into machine control data for three typical five-axis machine tools. The developed postprocessor was used in a five-axis machine to conduct a trial cut, and its performance was verified by using it in a coordinate measurement machine. Xie et al. [22] designed a three-DoF parallel kinematic mechanism with high orientational capability, which is crucial for the development of five-axis machine tools with hybrid architectures. Sangveraphunsiri and Chooprasird [23] designed a unique hybrid five-DoF manipulator based on an H-4 parallel mechanism with three translational movements, one rotational movement (orientation angle), and a single-axis rotating table. Li et al. [24] proposed a five-axis hybrid robot for friction stir welding. This robot comprised a 2-SPR-RPS parallel mechanism (with one translation DoF and rotational DoF) and two gantries. In the 2018–2019 years, research was conducted on the application of hybrid robots (parallel–serial manipulators) [21,25] in diverse types of machining and different domains.

In order to ensure that the horizontal parallel RPFMT designed in this study is applicable to diverse machining settings, the Siemens NX commercial software was incorporated into this tool. The Autodesk Inventor design software was used to draw a 3D machined workpiece, which was introduced into the Siemens NX CAM software. A suitable cutting tool was selected to calculate the five-axis NC machining code for a complex convex surface. The NC codes were input into the LabVIEW software for five-axis postprocessing conversion. Before running the postprocessing program in LabVIEW, the coordinates of the origin of the workpiece were determined. The distance between the workpiece and the upper platform’s center (∆*x* and ∆*y*) was measured and input into the postprocessing conversion program for computation. The *X*-coordinate, *Y*-coordinate, and *Z*-coordinate of the converted NC codes of the horizontal parallel RPFMT were extracted and imported into the Siemens NX software for verification. After these coordinates were confirmed to be correct, the machining points were converted into the actuation values of the axes of the designed machine and input into a five-axis controller for machining.

The RPFMT designed in this study contained a horizontal three-axis parallel motion platform and a three-axis machine tools. The DELTA OPEN CNC controller was used for developing and integrating a human–machine interface and programmable controller to achieve automatic online measurement. The measured data could be displayed on the human–machine interface or stored in programmable logic controllers (PLC) and transmitted to the computer for computations. A concave workpiece was cut using rough and finishing machining methods to verify the accuracy of the designed RPFMT.

## 2. Inverse Kinematics of the Parallel Three-Axis Mechanism

**Figure 1:**
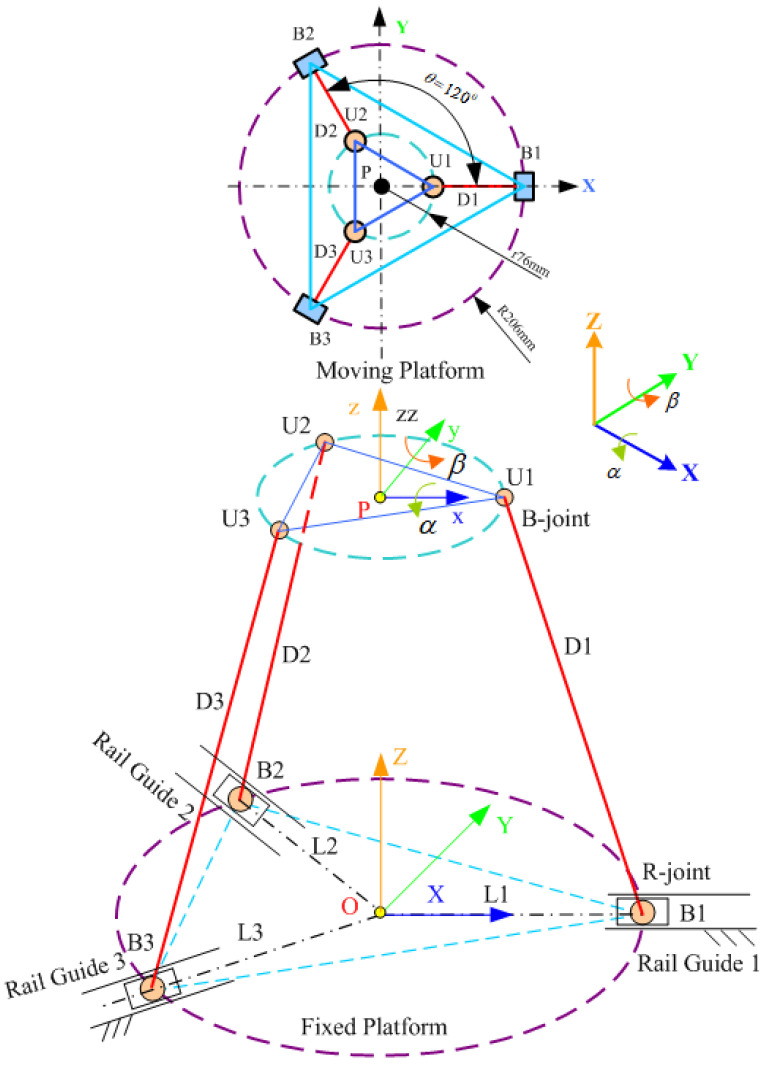
Horizontal parallel three-axis mechanism (3-PRS with intersecting rails).

First, the inverse kinematics of the three-axis mechanism was derived to control the trajectory, which is the extension from the center point of the moving platform (P) to the three actuation axes. Figure 1 depicts a schematic of the horizontal parallel three-axis mechanism.

The derived inverse kinematics can be used to convert the platform attitude into elongations along the three actuation axes. The positions of the connection points on the upper and lower platforms can be obtained by referring to Figure 1. The location of each connection point on the upper (moving) platform can be expressed as follows:

(1) UUi=r⋅[CθiSθi01]T; i=1~3

(2) BBi=r⋅[CθiSθi01]T

where r is the radius of the concave element circumscribed by the moving platform; $L_{i}$ is the displacement of the actuation axis on the fixed platform;

$$\theta_{i}=\frac{2\pi }{3}(i-1);\;i=1\sim 3;$$

The coordinates of the moving platform and the fixed platform’s coordinate system ($X_{B},Y_{B},Z_{B}$) are expressed as follows:

(3) UBi=[TBU]∗[UUi]; i=1~3

where

(4) TBU=[RBU00Z0001]

The parameter *Z* represents the distance between the lower platform’s center and the upper platform’s center along the *Z*-axis. The coordinates of the moving platform are expressed in the coordinate system of the fixed platform B by using the roll–pitch–yaw angles and considering γ=0. Therefore, the rotation coordinate of the moving platform is expressed as follows:

(5) RBU=Ry,β⋅Rx,α=[cosβsinβ⋅sinαsinβ⋅cosα0cosα−sinα−sinβcosβ⋅sinαcosβ⋅cosα]

Therefore, on the basis of Equation (3), the connection points on the moving platform are converted into the coordinate system of the fixed platform as follows, and *S* and *C* denote sin () and cos (), respectively.

(6) UBi=RBU⋅BUi=[UBixUBiyUBiz]=[r⋅Cθi⋅Cβ+r⋅Sθi⋅Sβ⋅Sαr⋅Sθi⋅CαZ−r⋅Cθi⋅Sβ+r⋅Sθi⋅Cβ⋅Sα]

The connecting rods are expressed in the vector form as follows:

(7) $${\vec{D}}_{i}=\vec{U_{i}B_{i}}$$

The length of each connecting rod is determined as follows:

(8) Di=UBi−BBi

where $D_{i}$ is the connecting rod length (fixed length).

(9) Di2=(UBix−BBix)2+(UBiy−BBiy)2+(UBiz−BBiz)2

where BBiz=0.

(10) Di2=(UBix−BBix)2+(UBiy−BBiy)2+UBiz2

The expansion of the brackets in Equation (10) results in the following equation being obtained:

(11) Di2=UBix2−2⋅UBix⋅BBix+BBix2+UBiy2−2⋅UBiy⋅BBiy+BBiy2+UBiz2

The expression BBix2+BBiy2=Li2 is substituted into Equation (11) to obtain the following equation after rearrangement:

(12) Li2−2⋅(UBix⋅BBix+UBiy⋅BBiy)+UBix2+UBiy2+UBiz2−Di2=0

By substituting Equation (2) into Equation (12), the following equation is obtained:

(13) Li2−2⋅(UBix⋅Cθi+UBiy⋅Sθi)⋅Li+UBix2+UBiy2+UBiz2−Di2=0

Equation (13) is solved to obtain the following two solutions:

(14) $$L_{i}=\frac{-b_{i}+\sqrt{b_{i}^{2}-4\cdot c_{i}}}{2}(i=1\sim 3)$$

$$L_{i}=\frac{-b_{i}-\sqrt{b_{i}^{2}-4\cdot c_{i}}}{2}\;(\mathrm{negative}\;\mathrm{discrepancy})$$

where

bi=−2⋅(UBix⋅Cθi+UBiy⋅Sθi); ci=UBix2+UBiy2+UBiz2−Di2

### Five-Axis Postprocessing Formula for the RPFMT

**Figure 2:**
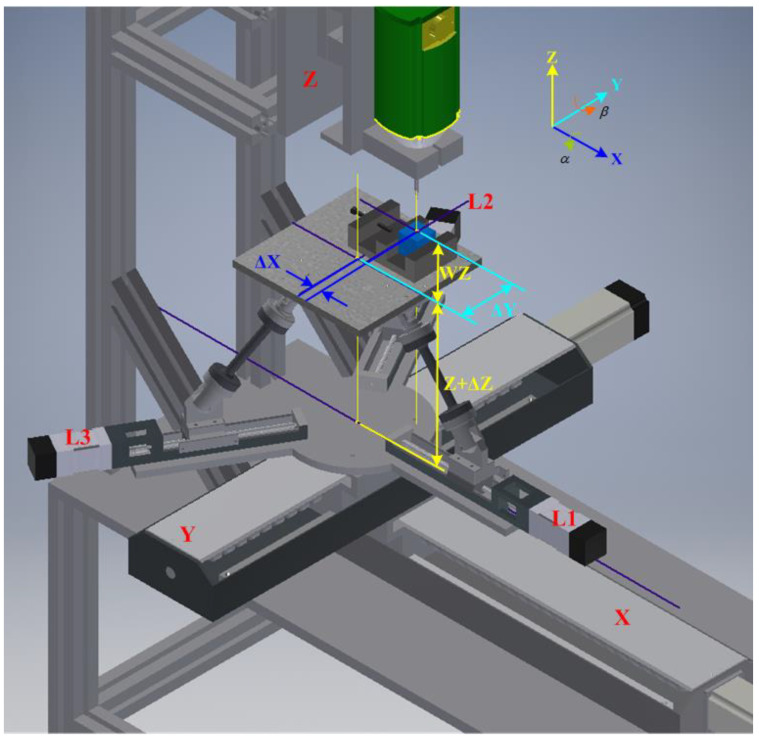
Spatial diagram of a five-axis RPFMT.

The five-axis postprocessing equation was derived from Figure 2 and is expressed as follows.

(15) TUW=[100ΔX010ΔY001AWZ0001]

where TUW is the transposed matrix of the coordinates of the centers of the workpiece and upper platform, Δ*X* is the distance between the workpiece’s center and the upper platform’s center along the *X*-axis, and Δ*Y* is the distance between the workpiece’s center and the upper platform’s center along the *Y*-axis.

The coordinates of the workpiece’s center were obtained using a touch-point sensor in this study.

(16) *AWZ* = Δ*Z* + *WZ*

where *WZ* is the height of the workpiece, which is calculated by adding the height of the workpiece with the platform plane as 0 (30 mm) and the height of the clamp (10 mm), and Δ*Z* is the height ascended by the platform (12 mm).

(17) $$T=\left[\begin{array}{cccc} C\beta & S\alpha \cdot S\beta & C\alpha \cdot S\beta & ΕX\\ 0 & C\alpha & -S\alpha & ΕY\\ -S\beta & S\alpha \cdot C\beta & C\alpha \cdot C\beta & ΕZ\\ 0 & 0 & 0 & 1 \end{array}\right]$$

(18) EX=ΔX·cosβ+ΔY·sinα·sinβ+AWZ·cosα·sinβ

(19) EY=ΔY·cosα+AWZ·(−sinα)

(20) EZ=ΔX·(−sinβ)+ΔY·sinα·cosβ+AWZ·(1−cosα·cosβ)

## 3. Experimental Setup

### 3.1. Automatic Measurement System

**Figure 3:**
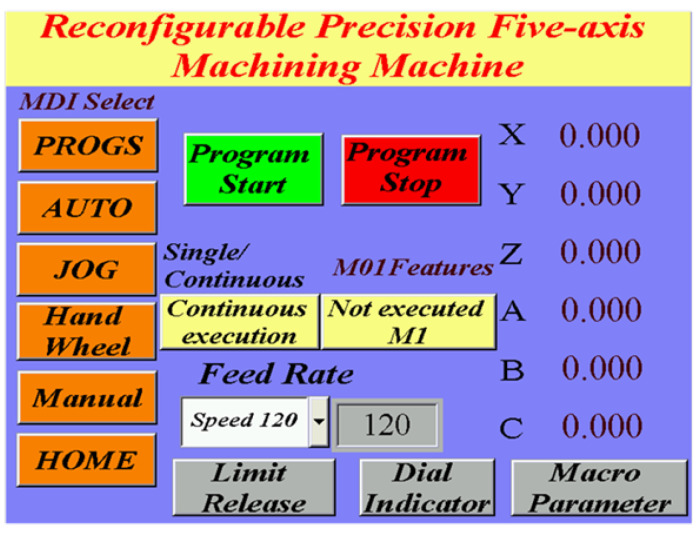
Operation of the DOP-B07E515 human–machine interface.

**Figure 4:**
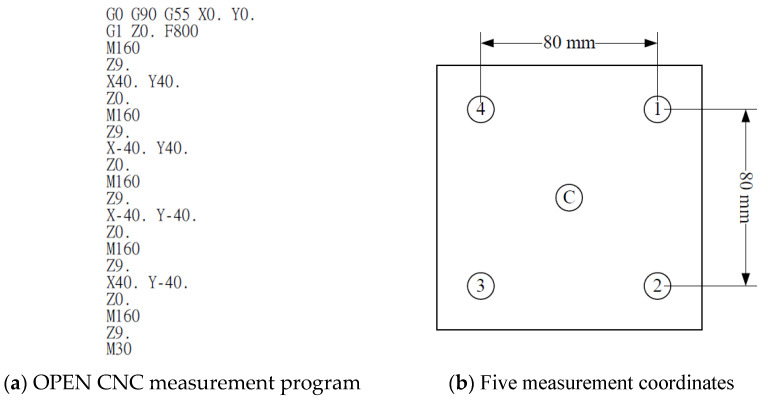
OPEN CNC measurement program and five measurement coordinates.

**Figure 5:**
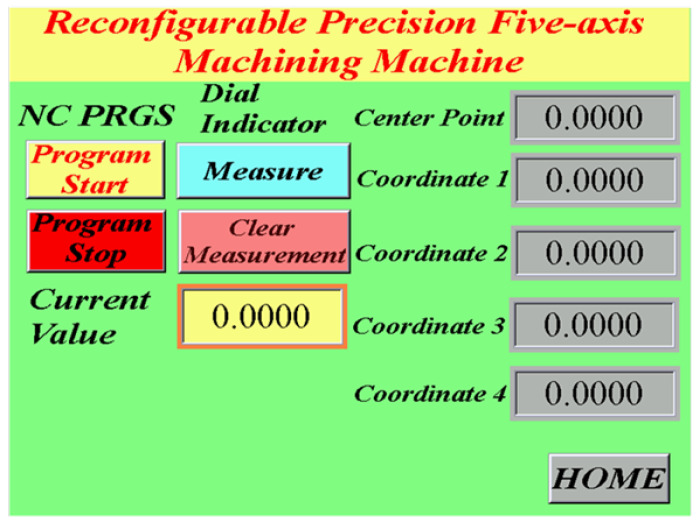
Real-time display of measured values on the human–machine interface.

**Figure 6:**
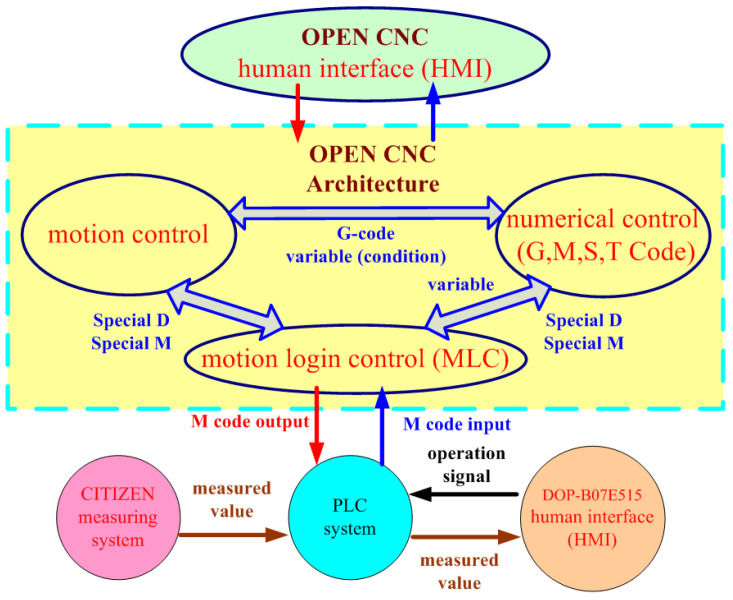
Flow diagram of the measurement based on OPEN CNC.

The main advantage of five-axis machine tools is that they can be used for the high-efficiency and precise machining of complex convex surfaces. The machining quality is further enhanced if these tools are equipped with a real-time online measurement system. Such systems are currently available in the market; however, they are costly and unsuitable for parallel five-axis machine tools. Therefore, an automatic online measurement system was developed in this study by integrating a human–machine interface and programmable controller into the self-developed OPEN CNC controller of the designed RPFMT (Figure 3, Figure 4 and Figure 5). According to user needs, the designed system can be controlled through the interface of the OPEN CNC controller or human–machine interface. The automatic online measurement system, which is used to control the designed machining tool, was developed using a DELTA OPEN CNC NC50EM controller. In this system, a DELTA DOP-B07E515 human–machine interface, DELTA DVP-PS02 programmable controller, and CITIZEN electronic comparison probe (IPD-B535) are integrated to achieve automatic online measurement. Before measurement, the continuous measurement of the coordinates or curves to be measured was edited in the CNC machining program. For user convenience, the measurement system could be operated with an OPEN CNC system interface or a DELTA DOP-B07E515 human–machine interface (Figure 3). Figure 4 presents the OPEN CNC measurement program (Figure 4a) and five measurement coordinates (Figure 4b). The measured values were displayed on the DELTA DOP-B07E515 human–machine interface (Figure 5). Figure 6 shows the flow diagram for the measurement based on OPEN CNC. A video on YouTube served as a reference in the experiment conducted on the automatic measurement system [26].

### 3.2. Experimental Framework

**Figure 7:**
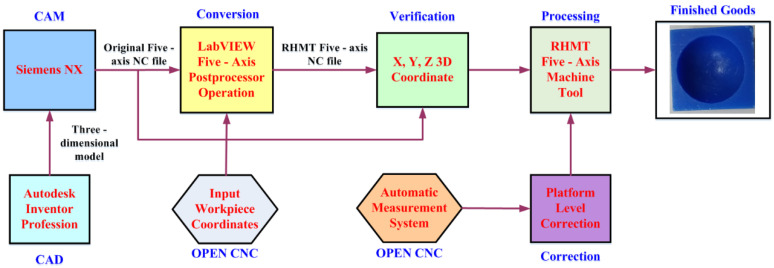
Flow diagram of the experimental process.

**Figure 8:**
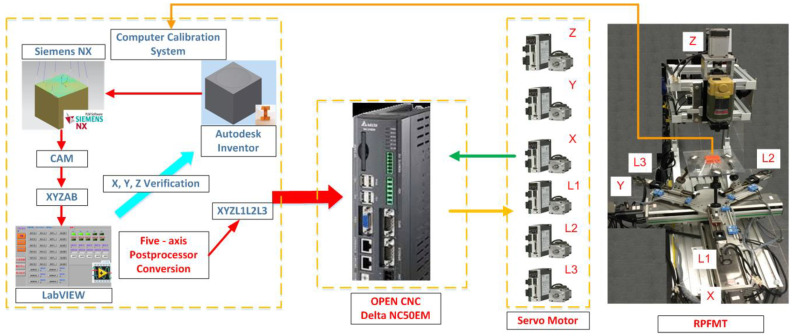
Architecture of the RPFMT.

Figure 7 displays the flow diagram of the experimental process in this study. First, a 3D machining workpiece was drawn using Autodesk Inventor and introduced into Siemens NX to compute the NC codes of the five axes. The NC codes of the horizontal parallel five-axis machine tools were obtained using the postprocessing program of LabVIEW. Before running this program, the coordinates of the origin of the machined workpiece were determined. The distance between the workpiece and the upper platform’s center (∆*x* and ∆*y*) was measured and input into the conversion program for computation. The *X*, *Y*, and *Z* values of the obtained five-axis NC codes of the RHMT were extracted, and 3D coordinates were plotted. These values were compared with those corresponding to the initial five-axis NC code. After being confirmed as correct, the aforementioned values were input into the DELTA OPEN CNC NC50EM controller to perform machining safely. The horizontal calibration of the three-axis platform of the RHMT was performed using the automatic measurement system. Figure 8 illustrates the architecture of the RHMT.

## 4. Results and Discussions

**Figure 9:**
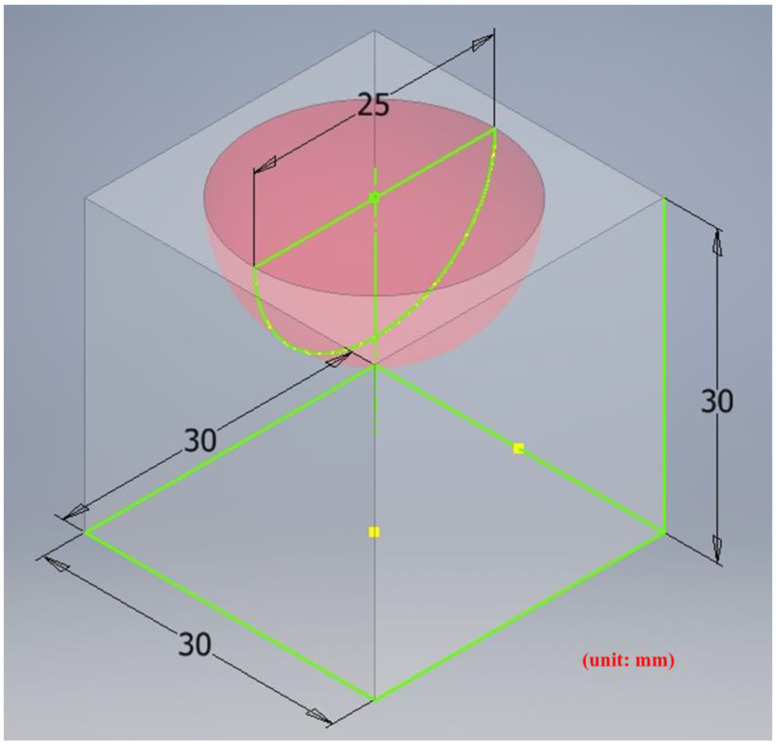
The dimension of concave circle processing.

A general five-axis machine tools comprises three lateral movement axes (the *X*-axis, *Y*-axis, and *Z*-axis) and two rotation axes (the *A*-axis and *B*-axis, *B*-axis and *C*-axis, or *A*-axis and *C*-axis). The designed RPFMT contained a three-axis (*XYZ*) machining tool and a horizontal parallel three-axis motion platform (*Z*, *A*, *B*). Therefore, in the Siemens NX software, the five-axis tool was set to be orthogonal, which corresponds to the spindle SBSA-type. A solid carbide R2 ball nose cutter was selected as the cutting tool, the “toward point” function was adopted, and the spindle speed was 1200 rpm (Revolutions per Minute). The main characteristics of the ball nose cutter: the number of cutting edges was 2, the tool diameter was 4 mm, the helix angle was 35 degrees, and the total length was 50 mm. The shank was designed to swing on a point 150 mm above the *Z*-axis to enable the machining process to adapt to the changes in the size of the machining center’s working space [6]. The side cutting edge was selected as the cutting tool mainly because the center point of the ball nose cutter is only a quiescent point without a cutting edge and is therefore unsuitable as a machining point. Figure 9 shows the dimension drawing for concave machining [9]. The machine in this study belongs to precision light machining, so the cutting material is wax block. In the experiment, workpieces were cut through rough and finishing machining methods to verify the accuracy of the designed RPFMT.

### 4.1. Rough and Finishing Machining of the Initial Concave Element

**Figure 10:**
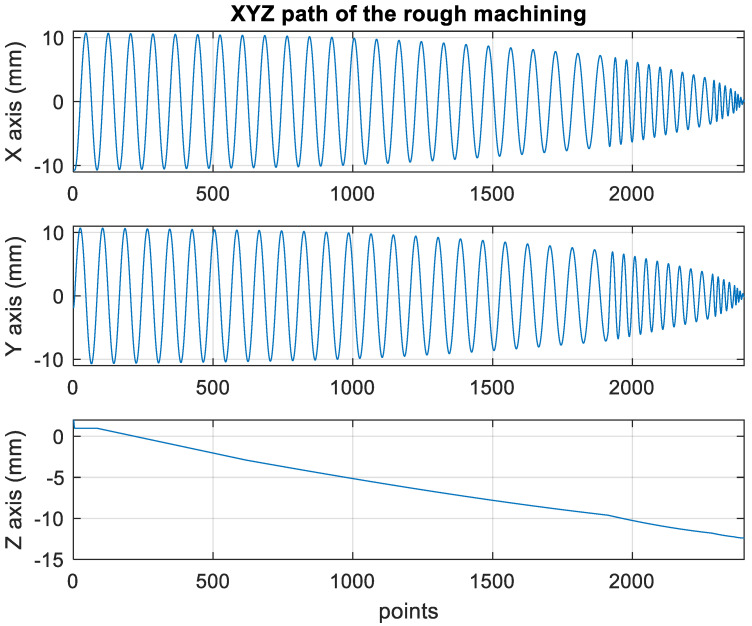
XYZ path of original rough machining program.

**Figure 11:**
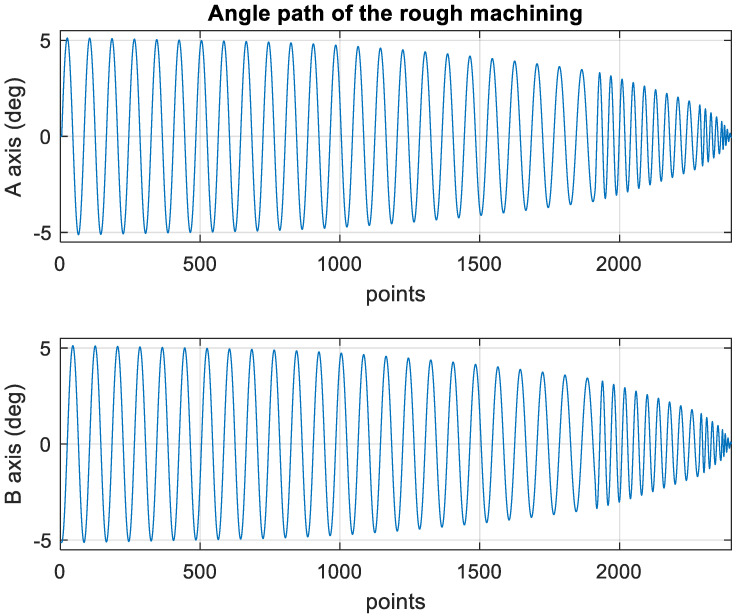
Angle path of the original rough machining program.

**Figure 12:**
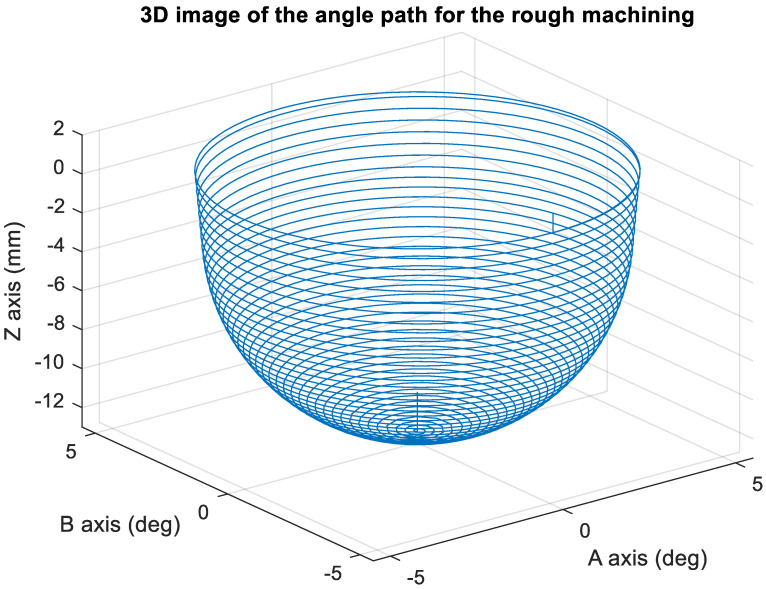
Three-dimensional image of the angle path with Z axis for the original rough machining program.

**Figure 13:**
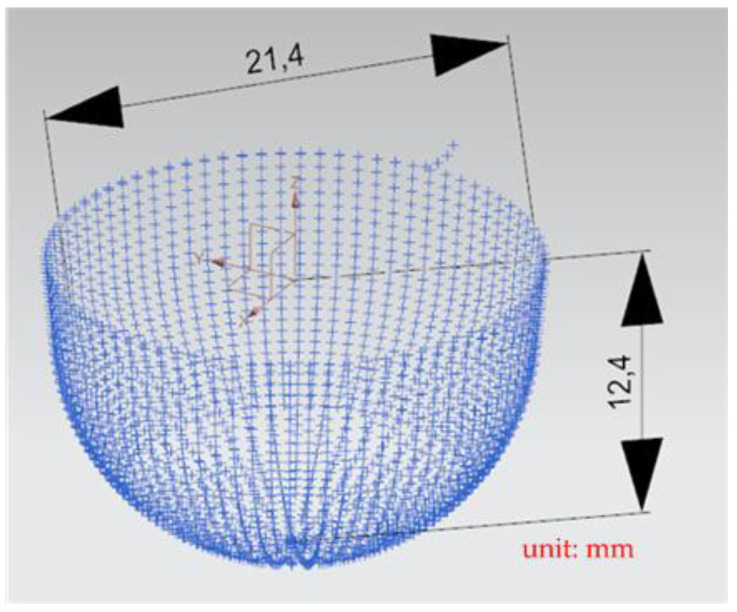
Spatial diagram of original concave circle roughing machining.

Figure 10 presents the *X*, *Y*, and *Z* values obtained for the rough machining of the initial concave element by using Siemens NX. Figure 11 presents the values of *A* and *B* for the cutting tool deflection angle obtained for rough machining. Figure 12 shows a 3D image of the *Z*-axis-oriented AB path at the cutting tool deflection angle. For the safety of the machining process and the accuracy of the five-axis software program, the values of *X*, *Y*, and *Z* in the five-axis NC codes were extracted and imported into the Siemens NX software, and a spatial diagram containing 2409 cutting points was drawn (Figure 13). The ruler in the Siemens NX software was used to measure the distance between two points. The maximum diameter of a concave circle was 21.4 mm, and a machining allowance was 12.4 mm.

**Figure 14:**
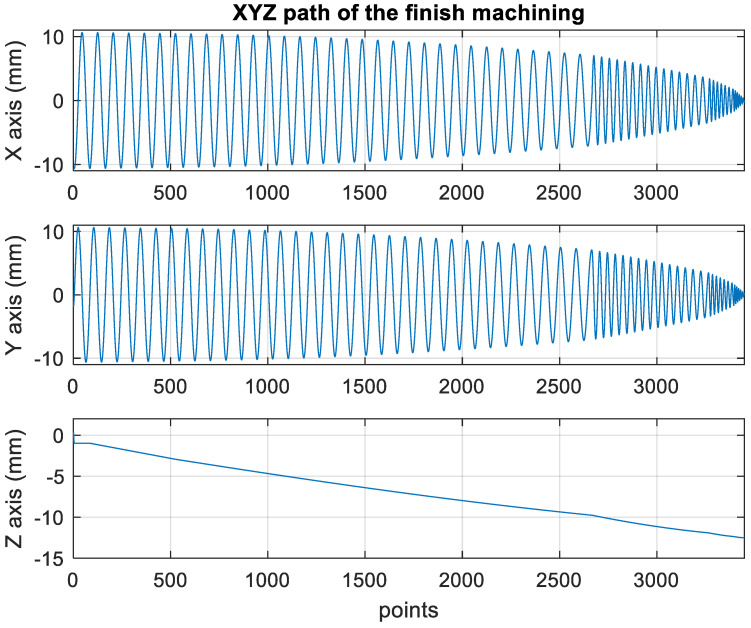
XYZ path of original finish machining program.

**Figure 15:**
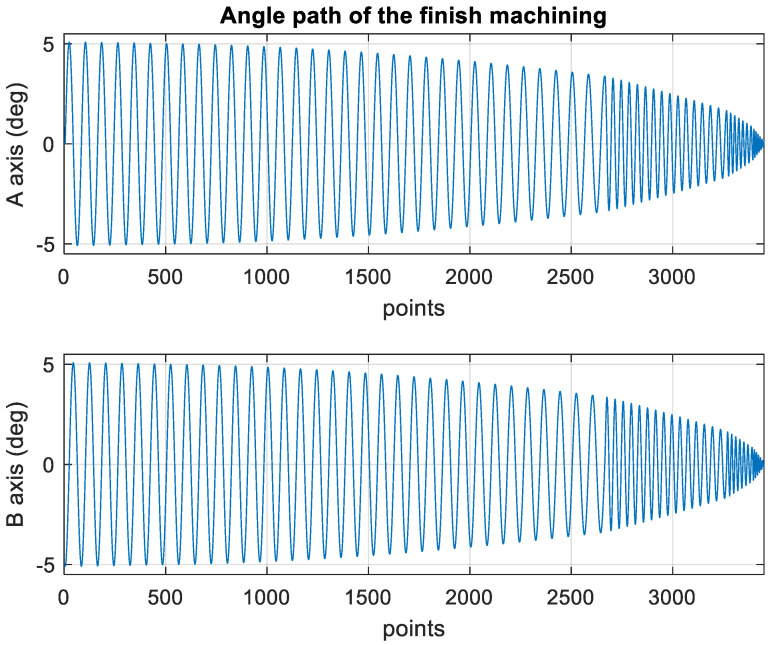
Angle path of the original finish machining program.

**Figure 16:**
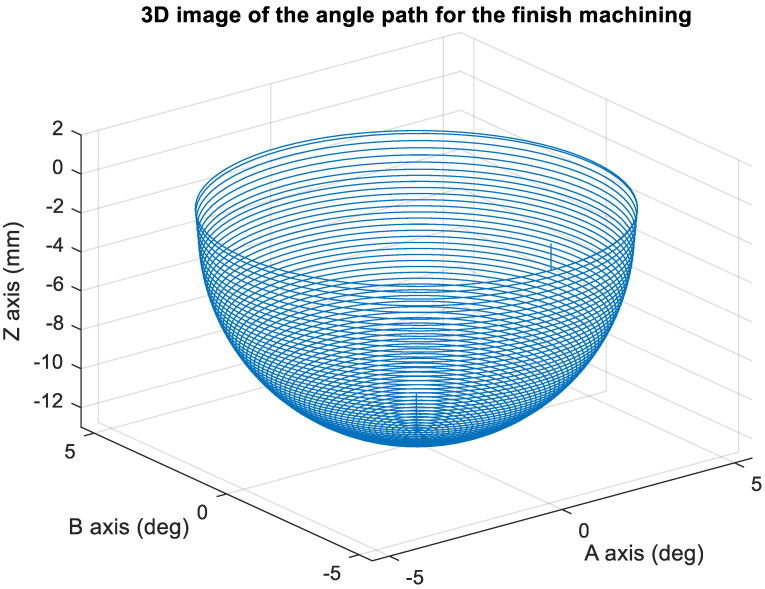
Three-dimensional image of the angle path with Z axis for the original finish machining program.

**Figure 17:**
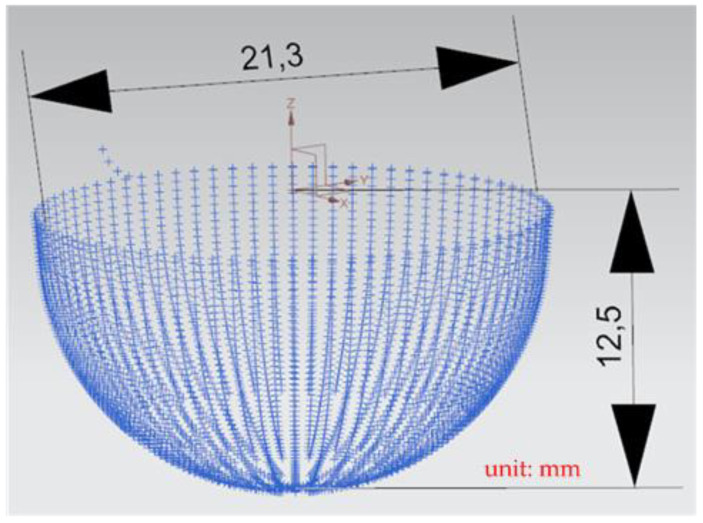
Spatial diagram of original concave circle finish machining.

Figure 14 presents the *X*, *Y*, and *Z* values obtained for the finishing machining of the initial concave element by using Siemens NX. Figure 15 presents the values of A and B for the cutting tool deflection angle obtained for finishing machining. Figure 16 shows a 3D image of the *Z*-axis-oriented AB path at the cutting tool deflection angle. Figure 17 depicts a spatial diagram of 3463 cutting points of finishing machining for the initial concave element. The maximum diameter of a concave element was 21.3 mm, and a machining allowance was 12.5 mm.

### 4.2. Converted Concave Circle Rough and Finishing Machining

The coordinates of the workpiece’s origin were measured before running the postprocessing conversion program for the horizontal parallel five-axis machine tools. The distance between the workpiece and the upper platform’s center (∆*x* and ∆*y*) was measured and input into the conversion program in LabVIEW. The workpiece’s origin coordinates (∆*x* 5.342, ∆*y* 2.346) were obtained through measurement with a touch-point sensor (VPS-301, MISUMI).

**Figure 18:**
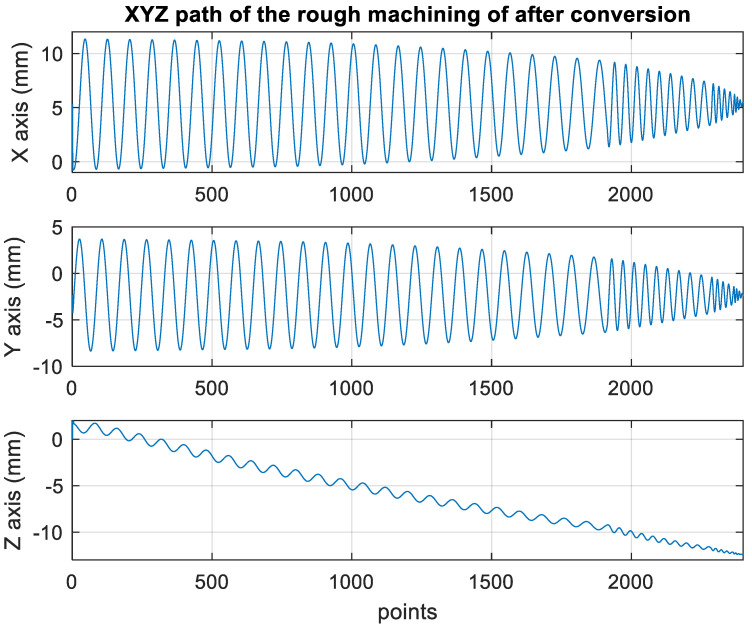
The path of the XYZ axis after converting the rough machining program.

**Figure 19:**
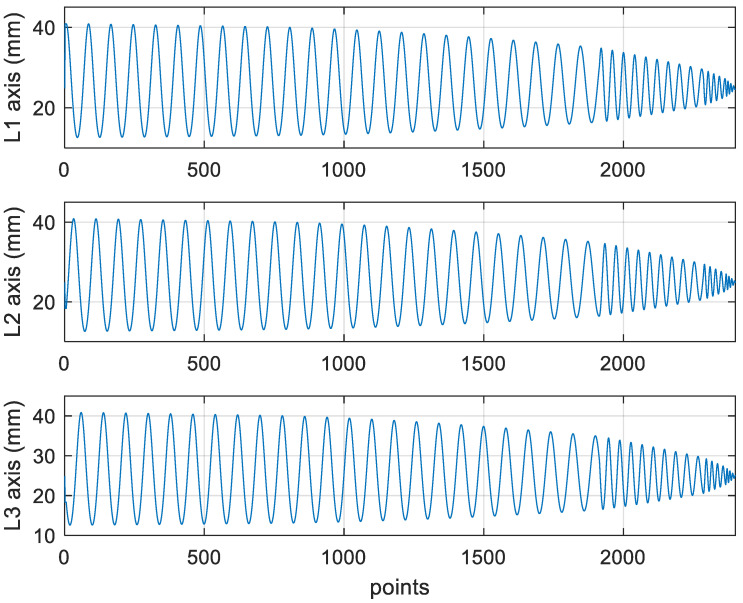
The lengths of the parallel three-axes after converting the rough machining program.

**Figure 20:**
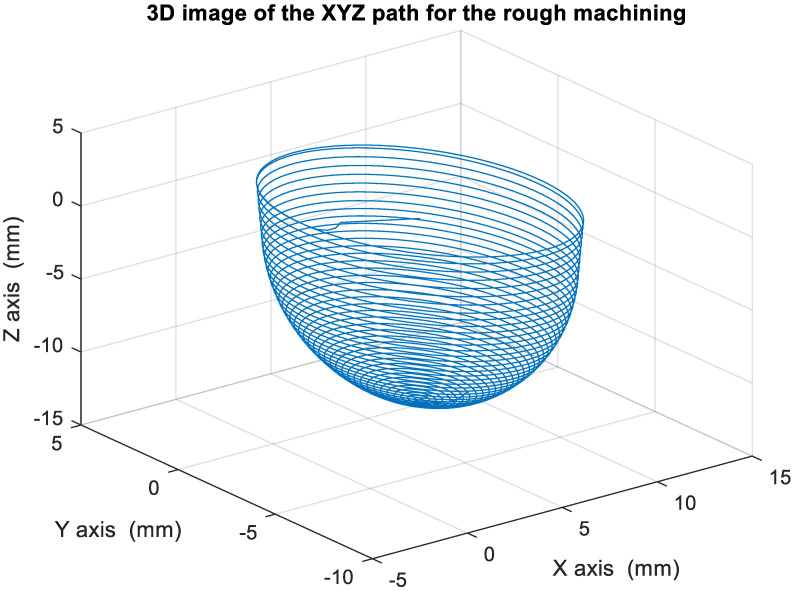
Three-dimensional image of the XYZ path after converting the rough machining program.

**Figure 21:**
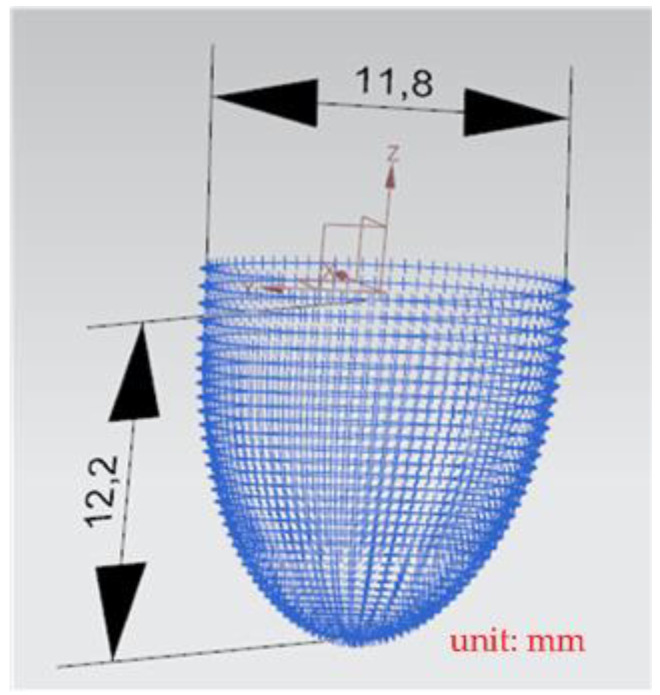
Spatial diagram of the converted concave circle rough machining.

Figure 18 depicts the *X*, *Y*, and *Z* values obtained for the rough machining of the final concave element by using Siemens NX. Figure 19 presents the displacement values obtained for the three axes of the platform under rough machining after running the conversion program. Figure 20 depicts a 3D image drawn on the basis of the *X*, *Y*, and *Z* values presented in Figure 18. Figure 21 shows a spatial diagram of the converted 2409 cutting points for rough machining. The maximum distance between the two points was 11.8 mm, and the machining allowance was 12.2 mm.

**Figure 22:**
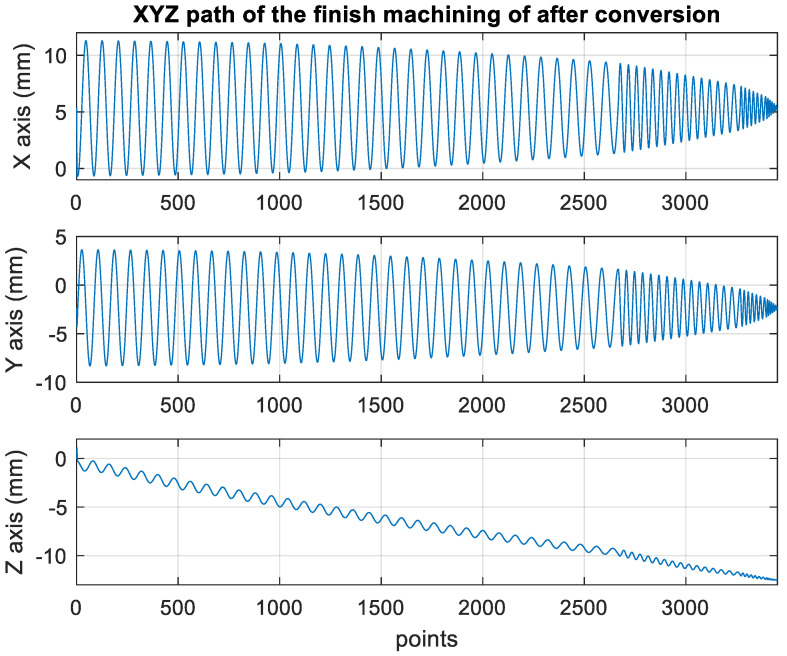
The path of the XYZ axis after converting the finish machining program.

**Figure 23:**
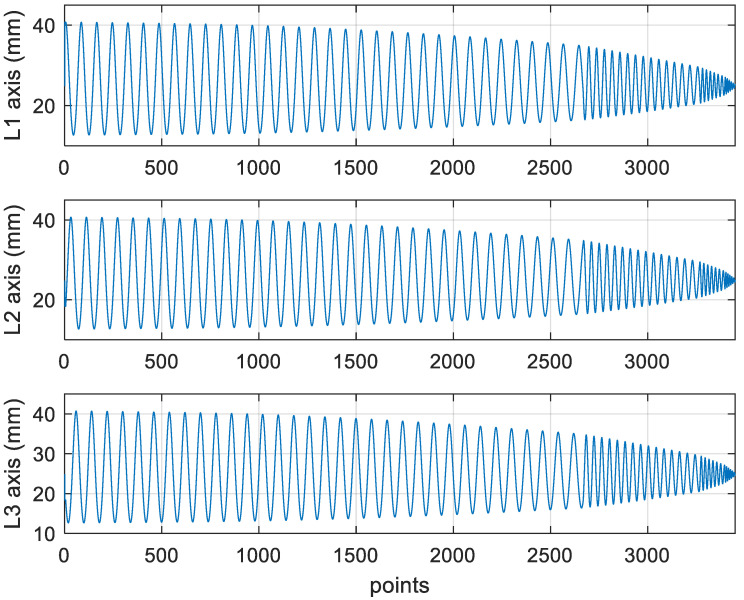
The lengths of the parallel three-axes after converting the finish machining program.

**Figure 24:**
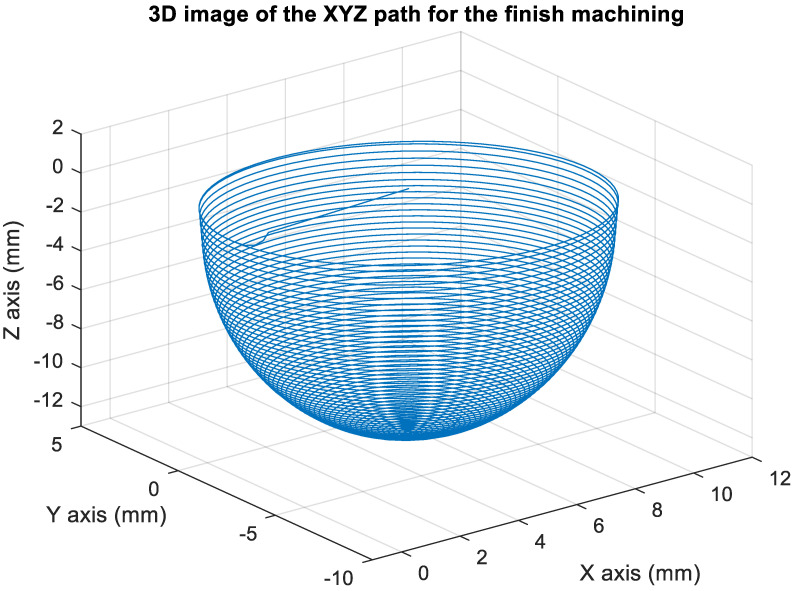
Three-dimensional image of the XYZ path after converting the finish machining program.

**Figure 25:**
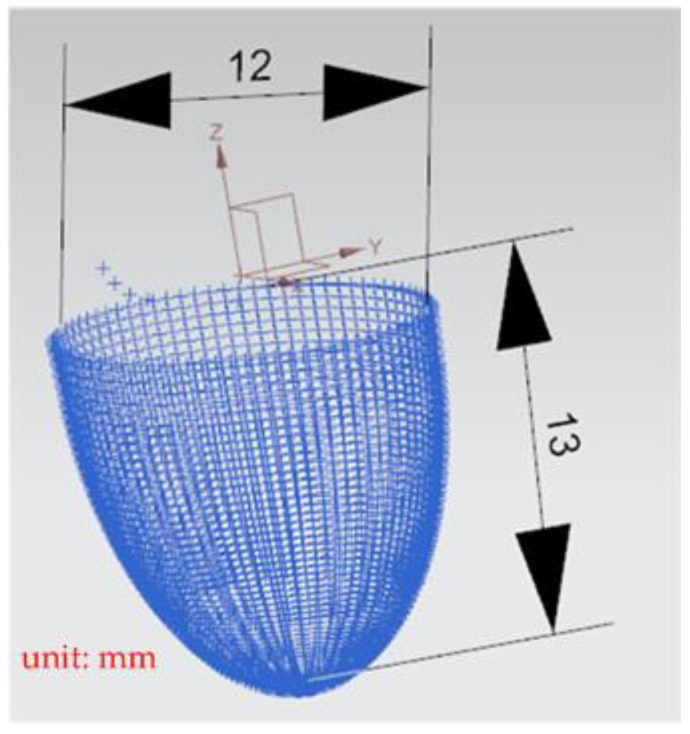
Spatial diagram of the converted concave circle finish machining.

Figure 22 shows the converted *X*, *Y*, and *Z* values obtained for the finishing machining of the final concave element by using Siemens NX. Figure 23 presents the motion values obtained for the three axes of the platform under finishing machining after running the conversion program. Figure 24 displays a 3D image drawn on the basis of the *X*, *Y*, and *Z* values displayed in Figure 22. Figure 25 shows a spatial diagram of the converted 3463 cutting points for finishing machining. The maximum distance between two points was 12 mm, and a machining allowance was 13 mm. A YouTube video served as a reference in the finishing machining experiment [27].

### 4.3. Discussions

In order to ensure that excess material could be removed, the cutting height was set as 2 mm above the plane in rough machining (Figure 10); thus, a machining allowance was observed for rough machining (13 mm, as shown in Figure 21). Figure 20 and Figure 24 indicate that the centers of the two cut concave elements deviated from (5.342, 2.346). Therefore, these elements were skewed. Figure 10 and Figure 14 indicate that during the five-axis machining of the initial element, the values of *X* and *Y* gradually decreased from ±10 mm to 0 as the *Z*-axis descended. Figure 18 and Figure 22 present the converted values of *X* and *Y* and indicate that these values are considerably reduced. The main reason for the aforementioned phenomenon was that the three-axis platform rotated (Figure 19 and Figure 21). For precision cutting of concave circles, the *X* and *Y* values should be reduced. The maximum diameter for rough machining reduced from 21.4 mm (Figure 13) to 11.8 mm (Figure 21), and the maximum diameter for finishing machining was reduced from 21.3 mm (Figure 17) to 12 mm (Figure 21). The maximum depth for rough machining increased from 12.4 mm (Figure 13) to 13 mm (Figure 21), whereas that for finishing machining decreased from 12.5 to 11.6 mm. The aforementioned results are comparable to those of [9], in which a parallel five-axis machine tools with an R5 ball nose cutter was adopted to cut a concave element. Because of the relatively large size of the R5 ball nose cutter, only 352 rough machining points were obtained in [9]. In [9], the cut diameter was 15.53 mm, and the machining allowance was 12.4 mm.

## 5. Conclusions

In this study, a horizontal parallel three-axis motion platform and a three-axis machine tools were integrated to construct an RPFMT. The DELTA OPEN CNC NC50EM controller was also incorporated into this RPFMT. The designed RPFMT demonstrates the advantages of parallel mechanisms and possesses the continuous tracking ability of the three-axis machine tools. A DELTA DOP-B07E515 human–machine interface, DELTA DVP-PS02 programmable controller, and CITIZEN electronic comparison probe (IPD-B535) were integrated to develop an automatic online measurement system. The measured data can be displayed on the human–machine interface in real time or stored in PLC and transmitted to the computer of the system for computations. The adopted three-axis platform can rapidly calibrate the surface and conduct real-time workpiece dimension measurement, which ensures machining quality.

A precise machining quality requires not only the use of high-quality machines and controllers but also the use of computer-aided design (CAD)/computer-aided manufacturing (CAM) software. Tool position (CL) data files are generated from the toolpaths, and these CL data files can then be processed by a postprocessor to generate NC code. In this study, the NC code input is converted into five-axis postprocessing developed by LabVIEW software. The experimental roughing and finishing machining results were identical to those of the constructed 3D model; thus, the accuracy of the proposed system was validated. The proposed RPFMT can be applied for the high-precision machining of complex convex surfaces.

In the future, the five-axis program converter developed for RPFMT will be combined with tool position (CL) data files. Directly output the NC program that RPFMT can accept, and then input the DELTA OPEN CNC controller to control the machine tools to achieve a consistent operation process.

## Data Availability

Not applicable.
